# Comprehensive whole genome analysis of *Staphylococcus aureus* isolates from dairy cows with subclinical mastitis

**DOI:** 10.3389/fmicb.2024.1376620

**Published:** 2024-04-08

**Authors:** Ntelekwane George Khasapane, Jane Nkhebenyane, Zamantungwa Mnisi, Stanford Kwenda, Oriel Thekisoe

**Affiliations:** ^1^Department of Life Sciences, Centre for Applied Food Safety and Biotechnology, Central University of Technology, Bloemfontein, South Africa; ^2^Clinvet International, Study Operations, Bloemfontein, South Africa; ^3^Vectors and Vector-Borne Diseases Research Programme, Department of Veterinary Tropical Diseases, Faculty of Veterinary Science, University of Pretoria, Pretoria, South Africa; ^4^Sequencing Core Facility, National Institute for Communicable Diseases, National Health Laboratory Service, Johannesburg, South Africa; ^5^Unit for Environmental Sciences and Management, North-West University, Potchefstroom, South Africa

**Keywords:** subclinical mastitis, *Staphylococcus aureus*, virulence factors, antimicrobial resistance, whole genome sequencing

## Abstract

*Staphylococcus* species are the primary cause of mastitis in dairy cows across the world. *Staphylococcus aureus* has recently become a pathogen that is zoonotic and multidrug resistant. This study aimed to sequence whole genomes of 38 *S. aureus* isolates from 55 subclinical mastitis dairy cows of 7 small-scale farmers in the Free State Province, South Africa and document and their antimicrobial and virulence genes. The 38 isolates were grouped by the *in silico* multi-locus sequencing types (MLST) into seven sequence types (STs), that is (ST 97, 352, 152, 243) and three new STs (ST8495, ST8500, and ST8501). Thirty-three *S. aureus* isolates were divided into 7 core single-nucleotide polymorphism (SNP) clusters. Among the 9 distinct *spa-types* that were detected, *Spa-types* t2883 accounted for the majority of isolates at 12 (31.57%), followed by t416 with 11 (28.94%) and t2844 with 5 (13.15%). The data also revealed the identification of four (4) plasmids, with Rep_N (rep20) accounting for the majority of isolates with 17 (44.73%), followed by Inc18 (repUS5) with 2 (5.26%). These isolates included 11 distinct antimicrobial resistance genes and 23 genes linked to bacterial virulence. Surprisingly, no methicillin resistance associated genes were detected in these isolates. Genome data of the current study will contribute to understanding epidemiology *S. aureus* genotypes and ultimately aid in developing treatment and control plans to stop the spread of mastitis in the Free State province and South Africa as a whole.

## 1 Introduction

*Staphylococcus* species are known to cause acute to chronic infections/diseases that are related to increased morbidity to infected hosts such as humans and animals (Pattabhiramaiah and Mallikarjunaiah, 2023). There are about 53 species with 28 sub-species within the *Staphylococcus* genus with *S. aureus* being the main cause of persistent clinical and subclinical intramammary infections (IMI; Vanderhaeghen et al., 2015). The pathogenesis of *S. aureus* starts with teat colonization, through the intramammary space by either progressive colonization or changes in intramammary pressure caused by the milking machines (Maity et al., 2020; Vargová et al., 2023). In the mammary alveolus, *S. aureus* adheres to and enters mammary epithelial cells, which serve as the site for multiplication, eventually resulting in a chronic IMI (Maity et al., 2020). The molecular mechanisms underlying *S. aureus* IMI still need to be fully deciphered. Generally, bacteria sense host signals and adapt gene expression to match environmental conditions to cause infection (Elhawy et al., 2021). Numerous virulence factors (VFs) involved in adhesion, invasion, and host defense evasion are known and well-studied in *S. aureus*. These VFs are either found in bacterial genomes or are within the transmissible genetic elements in a bacterium (Naushad et al., 2019). The emergence of drug resistance is a serious challenge for mastitis control due to their extensive use in the dairy industry, for example, through the dry cow therapy, contributes to the emergence of antimicrobial determinants in *S. aureus*, including development of multiple drug resistance (Klibi et al., 2018; Liu et al., 2020; Omwenga et al., 2021; Gelalcha et al., 2022). Molecular epidemiology of staphylococcal species involved in IMI of dairy cattle have focused more on the use of multi-locus enzyme electrophoresis (MLEE), pulsed-field gel electrophoresis (PFGE), sequence-based typing schemes, such as multiple-locus sequence typing (MLST), multiple-locus VNTR (variable number of tandem repeats) analysis (MLVA), random amplification of polymorphic DNA (RAPD) analysis and staphylococcal protein A (*spa*) typing (Li, 2008). Although these methods are helpful in genetic analysis, their resolution is often not strong enough to reveal genetic differences between strains. However, whole-genome sequencing (WGS) of bacterial genomes has become the preferred method to understand microevolution, phylogenies, and inter and intraspecies differences (Sivakumar et al., 2023). Thus, in the current study we utilized WGS to characterize and understand the virulence and antimicrobial resistance mechanisms in *S. aureus* isolates of subclinical mastitis (SCM) dairy cows from small-scale farmers of the Free State Province.

## 2 Materials and methods

### 2.1 Sample selection and bacteriological analysis

The *S. aureus* isolates used in the present study were all obtained from milk samples collected from seven (7) small scale farms in three local Municipalities (Maluti-A-Phofung, Mantsopa and Setsotso) in the Free State Province of South Africa between year 2021–2022. The sample collection was conducted according to the guidelines of National Mastitis Council (2004). A total of 166 composite milk samples from individual cows were randomly screened for intramammary infection by means of somatic cell count (SCC) assay using flow cytometry (Mérieux NutriSciences, South Africa). Thereafter, based on the SCC results, only 220 individual quarters from 55 of 166 cows were subjected to California mastitis test (CMT) according to manufacturer’s instructions (DeLaval, South Africa) on farm and subsequently only 160 quarter milk samples were collected for another round of SCC and microbiological analysis. The CMT results were scored and interpreted as recommended by Karzis et al. (2017). Isolates were defined as *S. aureus* on the basis of being gram-positive cocci and catalase positive. Thereafter, matrix-assisted laser desorption ionization–time-of-flight mass spectrometry (MALDI-TOF MS) and gene sequencing were employed for further identification of the isolates as reported in our previous study (Ozbey et al., 2022; Khasapane et al., 2024). Furthermore, we performed phenotypic and genomic antimicrobial resistance based on disk-diffusion and PCR techniques (Khasapane et al., 2024). To evaluate the susceptibility of *Staphylococcus* isolates to widely used antimicrobial drugs, the single disk diffusion technique was utilized. Antibiotic discs (ThermoFischer, South Africa) comprising of gentamicin (10 μg), ampicillin (10 μg), tetracycline (30 μg), penicillin (10 μg), erythromycin (15 μg), ciprofloxacin (5 μg), and cefoxitin (15 μg) were utilized according to the Clinical Laboratory Standards Institute (CLSI, 2023) guidelines which are interpreted as intermediate (I), sensitive (S), and resistant (R).

### 2.2 Whole-genome sequencing

Whole genome sequencing of *S. aureus* isolates was conducted at the National Institute of Communicable Diseases (NICD) Sequencing Core Facility, South Africa. Briefly, multiplexed, paired-end libraries (2 × 150 bp) were prepared using the Illumina DNA Prep kit (Illumina, San Diego, United States), followed by sequencing on the Illumina NextSeq 2000 platform (Illumina, San Diego, United States) at 100× coverage.

### 2.3 Quality control and *de novo* assembly

Illumina paired-end reads were analyzed using the JEKESA bioinformatics pipeline v1.0^1^ including quality control, species identification and *de novo* assembly as previously described in Souvorov et al. (2018). Multilocus sequence typing (MLST) was performed using mlst v2.19.0 (--legacy -scheme saureus; Gurevich et al., 2013), based on traditional PubMLST typing schemes.^2^

### 2.4 Antimicrobial resistance prediction

Detection of antimicrobial resistance determinants was performed using a combination of three popular tools, namely, AMRFinderPlus (Feldgarden et al., 2021), ABRicate^3^ and staramr,^4^ by scanning the assembled contigs against ResFinder (Bortolaia et al., 2020), PointFinder^5^ and AMRFinderPlus databases. The outputs from these tools were summarized using HAMRonization.^6^

### 2.5 Phylogenetic analysis

Core genome SNPs (single nucleotide polymorphisms) were used to investigate the phylogeny and genetic relatedness of isolates. Briefly, whole genome alignments were performed using scapper^7^ and *Staphylococcus aureus* strain NCTC 8325 was used as a reference. Recombinant regions were removed using Gubbins v3.2.1(Croucher et al., 2015) and variable sites were obtained using snp-sites v2.5.1 (Page et al., 2016). Pairwise SNP distances were calculated using snp-dist v0.8.2^8^ and a normalized pairwise SNP distance matrix was used as input for the cluster analysis using the R software environment v4.2.1. Assignment of SNP clusters was achieved by a combination of K-means clustering implemented in the eclust function (factoextra package; v1.0.7; Kassambara, 2016) and custom functions written in R using a silhouette score and SNP cut-off of 0.5 and 20, respectively. Visualization of cluster heat maps was performed using the ComplexHeatmap package v.14.0 (Gu, 2022). IQ-TREE v2.0.3 (Minh et al., 2020) was used to generate a maximum-likelihood phylogenetic tree using the GTR + F + ASC + R4 with 1,000 bootstrap approximations using UFBoot2 (Hoang et al., 2018). The phylogenetic tree was visualized and annotated using Microreact.^9^

### 2.6 Core SNP cluster analysis and transmission network reconstruction

Pairwise SNP distances were calculated using snp-dist (v0.8.2; https://github.com/tseemann/snp-dists) using the final alignment file based on variable sites only. A normalized pairwise SNP distance matrix was used as input for the cluster analysis performed using the R software environment (version 4.2.1). Briefly, K-means clustering was done using default parameters in the eclust function from the factoextra package (v1.0.7; Kassambara, 2016), but with bootstrapping set to 500, and only core SNP clusters with a silhouette score ≥ 0.5 and SNP cut-off ≤ 25 were considered. Visualization of cluster heat maps was performed using the ComplexHeatmap package (v.14.0; Gu, 2022). A simple dendrogram showing the SNP clusters was generated using the fviz_dend function from the factoextra package. The minimum spanning tree was generated using the ape package in R (Paradis et al., 2004) based on pairwise SNP distances. Visualization of the minimum spanning tree was done using the visNetwork package (v2.2.2; https://datastorm-open.github.io/visNetwork/).

## 3 Results

### 3.1 Phenotypic antimicrobial resistance test

As reported in our previous study (Khasapane et al., 2024) the isolates showed resistance to penicillin 43/50 (86%), ciprofloxacin 40/50 (80%), vancomycin 39/50 (76%), and cefoxitin 26/50 (52%). Observed resistance against gentamycin, ampicillin, tetracycline, and erythromycin was 18/50 (36%), 14/50 (28%), 9/50 (18%), and 9/50 (18%), respectively.

### 3.2 Genome of mastitis-associated *Staphylococcus aureus* isolates

In the current study, whole genomes of 38 *S. aureus* isolates associated with bovine subclinical mastitis from Free State Province smallholder farms were sequenced. Table 1 provides an overview of genomic sequences.

**Table 1:** Shows the overall sequence types based on MLST, *spa-types*, and plasmid diversities.

Isolate no.	Strain name	Bio sample accession no.	MLST	No of raw reads	No. of Contigs	Coverage depth	N_50_ value (bp)	Genome length (bp)	*Spa* types	Plasmid
1-1	Pf01	SAMN37008033	97	2,455,396	31	143	193,876	2,750,926	t2883	*Rep_N (rep20)*
1-10	Pf02	SAMN37008034	97	2,995,734	48	157	156,217	2,829,526	t2883	*-*
1-3	Pf03	SAMN37008035	352	2,869,374	31	153	190,332	2,768,936	t730	*Rep_N (rep20)*
1-5	Pfogunf01	SAMN35674906	8,500	2,803,950	40	151	164,369	2,802,669	t416	*-*
1-6	Pf04	SAMN37008036	97	2,986,512	564	152	7,640	2,802,283	t2844	*-*
1-7	Phofung02	SAMN35674907	8,501	3,269,844	33	172	145,639	2,781,918	t189	*Inc18 (repUS5)*
1-8	Pf05	SAMN37008037	97	2,918,854	26	159	341,053	2,751,292	t2883	*Rep_N (rep20)*
1-9	Pf06	SAMN37008038	352	1,838,580	33	98	157,760	2,767,856	t730	*-*
2-10	Pf07	SAMN37008039	97	2,671,564	46	142	156,085	2,827,631	t2883	*-*
2-2	Pf08	SAMN37008040	97	2,526,344	43	135	189,485	2,828,626	t2883	*-*
2-3	Pf09	SAMN37008041	352	2,546,842	30	139	190,412	2,712,076	t2844	*-*
2-5	Phofung03	SAMN35674908	8,501	2,612,746	34	142	145,649	2,780,593	t189	*Inc18 (repUS5)*
2-7	Pf10	SAMN37008042	97	1,545,888	49	83	141,706	2,825,946	t2883	*-*
2-9	Pf11	SAMN37008043	97	2,110,296	51	113	140,217	2,829,158	t2883	*-*
3-1	setsoto01	SAMN35674909	8,500	2,908,804	42	124	122,277	2,799,261	t416	*Rep_N (rep20)*
3-10	setsoto02	SAMN35674910	8,500	3,126,264	41	157	176,781	2,799,455	t416	*Rep_N (rep20)*
3-4	setsoto03	SAMN35674911	8,500	2,755,396	31	149	181,801	2,757,166	t416	*Rep_N (rep20)*
3-5	setsoto04	SAMN35674912	8,500	2,196,012	32	116	341,110	2,788,663	t189	*Inc18 (repUS5)*
3-6	setsoto05	SAMN35674913	8,500	2,709,558	37	139	118,071	2,813,862	t416	*Rep_N (rep20)*
3-7	setsoto06	SAMN35674914	8,500	2,204,122	34	116	184,814	2,802,742	t416	*Rep_N (rep20)*
3-8	Se01	SAMN37008044	97	2,468,030	48	131	156,161	2,828,107	t2883	*-*
3-9	setsoto07	SAMN35674915	8,500	2,783,710	32	146	156,101	2,756,154	t416	*Rep_N (rep20)*
4-10	mantsopa01	SAMN35674916	8,500	2,801,174	31	149	210,386	2,802,954	t416	*Rep_N (rep20)*
4-2	Ma01	SAMN37008045	352	3,351,448	32	152	186,379	2,710,881	t2844	*-*
4-3	Ma02	SAMN37008046	352	2,782,766	27	65	190,324	2,710,723	t2844	*-*
4-4	mantsopa02	SAMN35674917	8,500	1,201,376	56	65	106,797	2,813,544	t4164	*Rep_N (rep20)*
4-5	Ma03	SAMN37008047	352	2,367,580	27	129	190,402	2,710,065	t4558	*-*
4-6	Ma04	SAMN37008048	97	2,549,736	30	139	341,053	2,750,257	t2883	*Rep_N (rep20)*
4-7	mantsopa03	SAMN35674918	8,500	2,630,238	39	140	225,079	2,815,611	t416	*Rep_N (rep20)*
4-8	Ma05	SAMN37008049	97	2,838,244	48	150	156,085	2,827,977	t2883	*-*
5-1	mantsopa04	SAMN35674919	8,500	1,768,784	38	89	181,707	2,802,171	t416	*Rep_N (rep20)*
5-10	mantsopa05	SAMN35674920	8,500	3,095,854	35	164	184,780	2,803,046	t4164	*Rep_N (rep20)*
5-2	Ma06	SAMN37008050	97	1,423,060	36	78	216,120	2,750,150	t2883	*Rep_N (rep20)*
						110				
5-3	Ma07	SAMN37008051	97	2,069,772	46		189,566	2,828,411	t2883	*-*
5-4	mantsopa06	SAMN35674921	8,500	2,474,622	32	135	181,811	2,756,261	t416	*Rep_N (rep20)*
5-5	Ma08	SAMN37008052	152	1,505,912	44	82	91,272	2,710,178	t355	*Inc18 (rep16)* and *Rep3 (rep5a)*
5-7	mantsopa07	SAMN35674922	8,495	2,589,764	35	142	185,000	2,717,325	t2844	*-*
5-9	Ma09	SAMN37008053	243	3,657,408	36	201	140,036	2,729,677	t21	*-*

### 3.3 Distribution of sequencing types, clonal complexes, spa-types and plasmids among all *Staphylococcus aureus* isolates from bovine SCM

**Figure 1:**
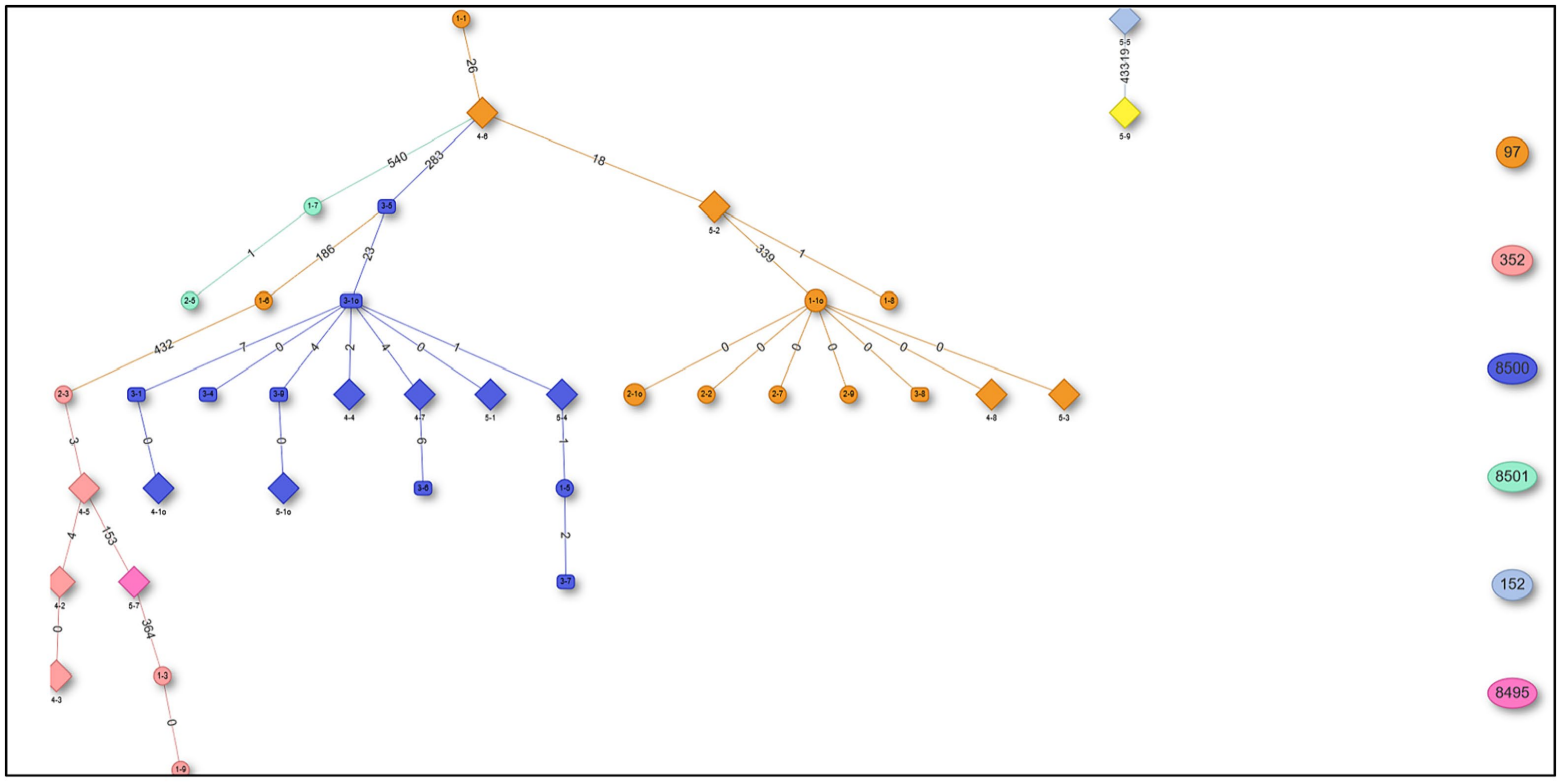
Minimum spanning tree (MST) showing sequencing types (STs) from 38 *Staphylococcus aureus* isolates.

**Figure 2:**
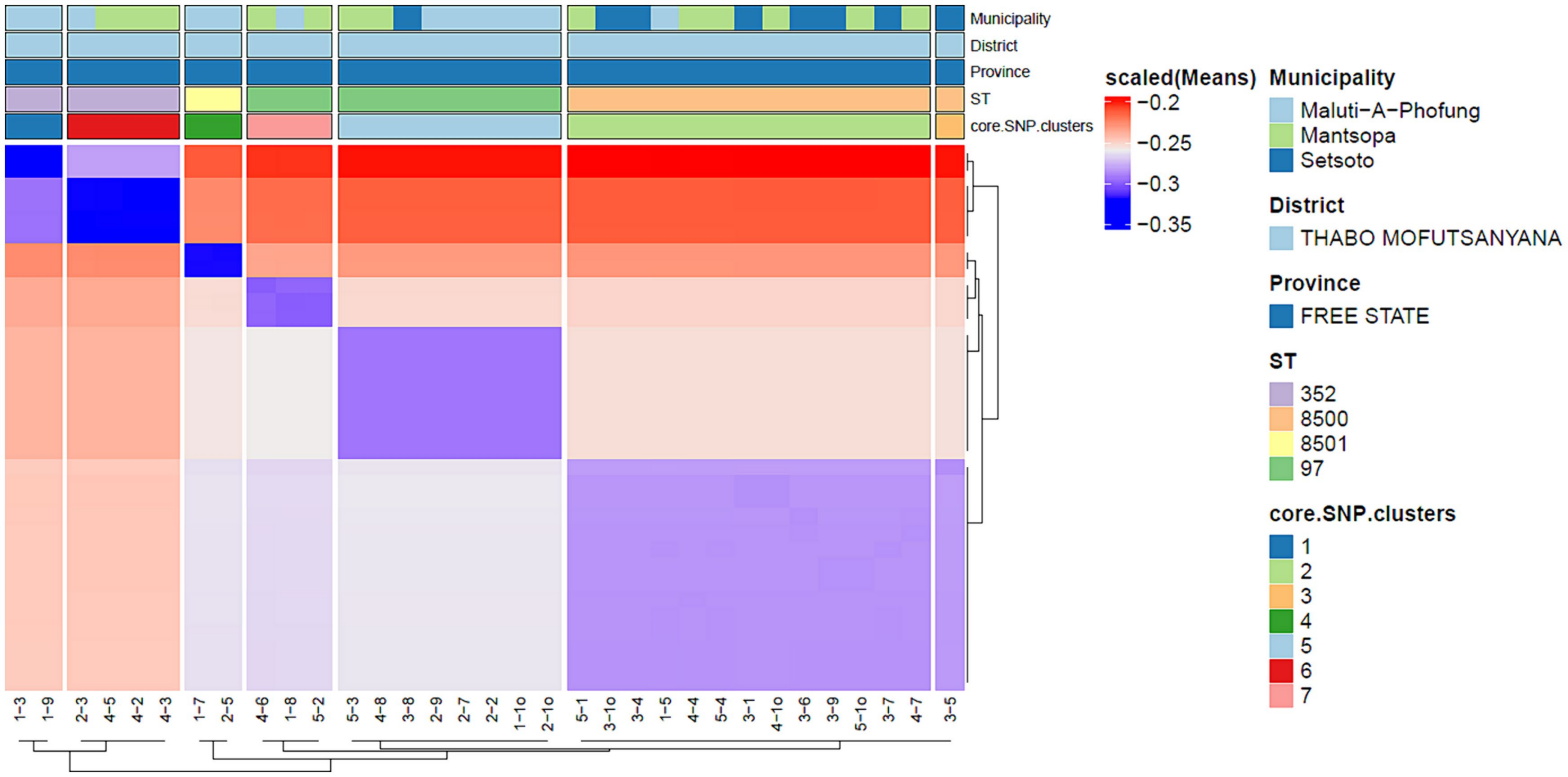
Depicts core genome SNP clusters.

The *in silico* MLST clustered the 38 isolates into 7 sequence types (STs; ST 97, 352, 152, 243) and 3 novel STs (ST8495, ST8500, and ST8501; Figure 1). Majority were assigned to the ST8500 *n* = 14 (36.8%) sequencing type, followed by ST97 *n* = 12 (31.5%), ST352 *n* = 7 (18.4%), ST8501 *n* = 2 (5.2%), while the latter STs [ST8495, ST152 and ST243] each had *n* = 1 (2.6%) isolates. Furthermore, whole genome sequencing analysis further revealed nine different spa-types of *S. aureus* and four types of plasmids from all isolates. Of all the *spa-types*, the t2883 accounted for most isolates with *n* = 12 (31.57%) followed by t416 with *n* = 11 (28.94%) and t2844 with *n* = 5 (13.15%). Interestingly, the study found 1 unknown *spa-type* t21 when the sequences were submitted to Ridom spa server,^10^ which we concluded to be novel spa type. While on the other hand plasmid Rep_N (rep20) was found in most of the isolates with *n* = 17 (44.73%), followed by Inc18 (repUS5) with *n* = 2 (5.26%) lastly one isolate (2.65%) had both plasmid rep16 and rep5a (Table 1; Figure 2).

### 3.4 Distribution of AMR and VFs within *Staphylococcus* isolates

**Figure 3:**
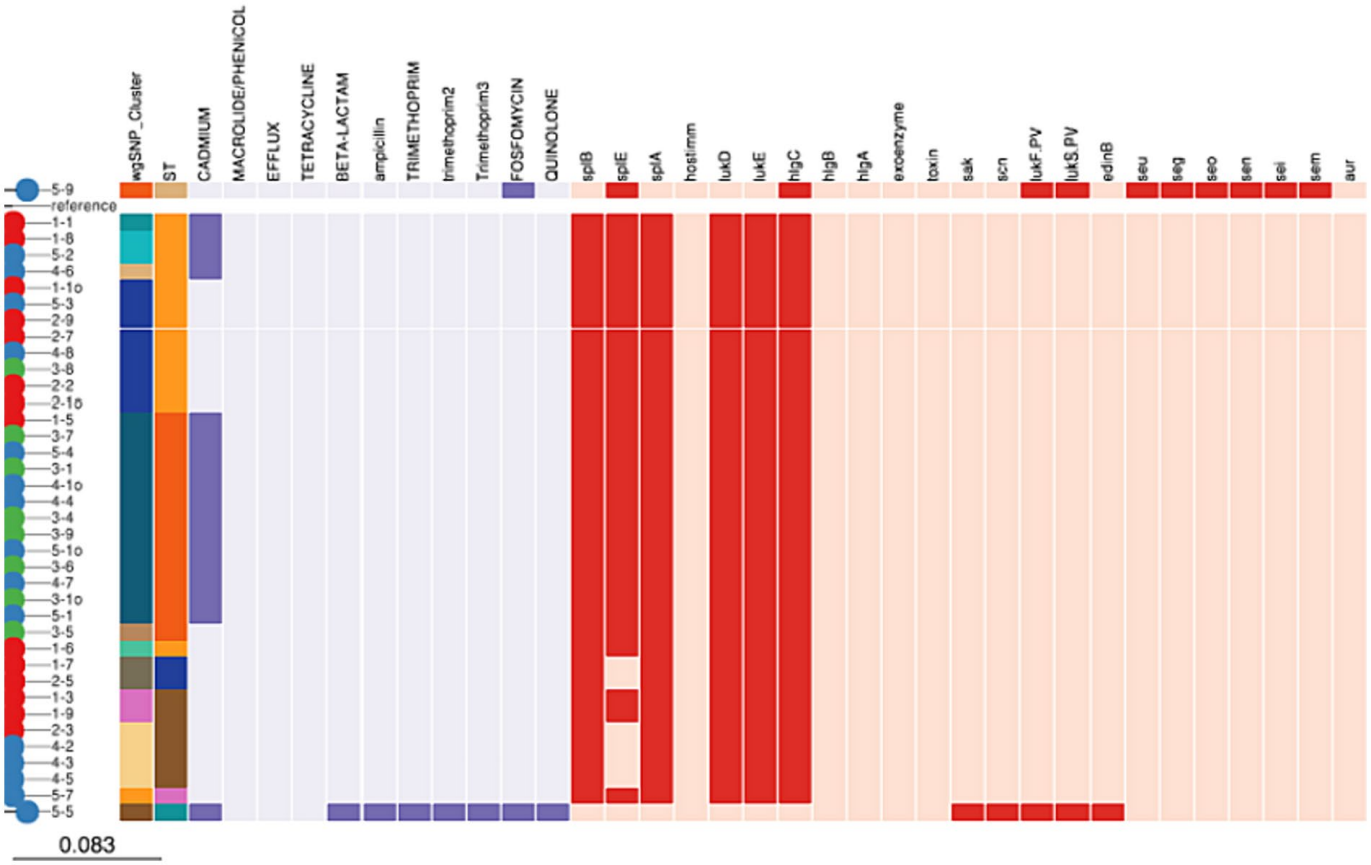
Distribution of antimicrobial resistance and virulence genes among *Staphylococcus aureus* isolates.

All isolates belonging to ST8500, ST97, ST352, and ST8501 contained *lmrS, mepA* and *tet* (38) genes which indicates resistance to were resistant to macrolide/phenicol, efflux and tetracycline, respectively. On the other hand, 18 (47.36%) of the isolates belonging 204 to ST8500 (31.57%), ST97 (10.52%) and ST152 (2.63%) carried *cadD* gene encoding for resistance against cadmium. Finally, Fosfomycin (*murA*) encoding gene was carried by 2 (5.26%) isolates belonging to ST152 and ST243, respectively. Beta-lactam, ampicillin (*blaI, blaR1*, and *blaZ*), trimethoprim (*dfrG* and *dfrG_1*), and quinolone (*parE*) genes were carried by one isolate each (2.63%) belonging to ST152 (Figure 3).

Our study further showed that all isolates (100%) belonging to ST8500, ST97, ST352, ST8501, ST243, ST152, and ST8495 carried *aur, HlgA*&*B* genes encoding for aureolysin factor, bi-component gamma hemolysin *HlgAB* subunit A and B, respectively. While 36 (94.7%) of the isolates belonging to ST8500, ST97, ST352, ST8500, and ST8495 carried LukD and *E* gene encoding for bi-component leukocidin LukE and D subunit D and E, respectively. Furthermore, *splB, splE*, and *splA* genes encoding for serine protease *A, B*, and *E* was carried by 36 (94.7%), 36 (94.7%) and 31 (81.5%) of all the isolates belonging to ST8500, ST97, ST152 and ST8495. Finally, 218 2 (5.26%) of the isolates contained *LukF.PV* and *LukS.PV* encoding for Panton-Valentine leucocidin only in ST152 and ST 243. While *seu, seg, seo, sen, sei*, *sem*, and *edinB* genes encoding for enterotoxins were detected in 1 (2.63%) of the isolates belonging to ST243 (Figure 3).

## 4 Discussion

Numerous investigations have examined potential variations in virulence gene profiles between *S. aureus* isolates from clinical and subclinical mastitis; however, no discernible differences have been found (Rocha et al., 2019; Naushad et al., 2020; Åvall-Jääskeläinen et al., 2021). According to Wellnitz and Bruckmaier (2012), mastitis is a dynamic phenomenon in which the microbe enters the mammary gland, causing leukocytes in the milk and mammary-gland epithelial cells to react and start the immunologic defense process. This causes a large number of neutrophils to migrate to the mammary gland and attempt to kill the microbial cells. In this study, we examined the presence of *S. aureus* associated with subclinical mastitis in dairy cattle in addition to associated antimicrobial resistance and virulence factors in 38 isolates.

Our identified nine *spa* types from *S. aureus* isolates and majority of them were assigned to t2883 (31.57%), followed by t416 (28.94%). Prior research has revealed a high diversity of *S. aureus* spa types in samples collected along dairy chains, including t2883 (Ben Slama et al., 2011; Boero et al., 2022). Interestingly, a study by Ben Slama et al. (2011) found the t2883 spa type in human hands that had come into touch with animals in Tunisia, suggesting that there may be a chance of transmission from human to animal or vice versa. In the current analysis, the t416 spa type was also discovered, and it appears to be dominant in all 38 isolates of *S. aureus*. The high prevalence of t416 spa type discovery in our investigation is more than that reported by Mora-Hernández et al. (2021), who found t416 in 6.06% of the *S. aureus* isolates from dairy cows in Mexico that had mastitis. The widespread use of antibiotics to treat *S. aureus*-caused human infections and cow mastitis is endangering public health since AMR is emerging among dangerous bacteria (Zigo et al., 2019). Finding the ARGs is necessary to evaluate the pathogenic potential of *S. aureus* during mastitis. Scanning genome sequences facilitates the identification of genetic components linked to virulence and antibiotic resistance (Paramasivam et al., 2023).

The phylogenomic study aligned with the known mechanism of zoonotic transmission of *S. aureus* (Silva et al., 2023). This may be explained by data indicating that, although many *S. aureus* lineages are non-specific, others are suited to colonize and infect particular host species (Schmidt et al., 2017).

It has been observed that majority of isolates across all clusters and STs were carrying the multidrug resistance genes *lmrS, mepA*, and *tet*, which encode for resistance against macrolide/phenicol, multidrug efflux MATE transporter *MepA* and tetracycline. Pu et al. (2021) carried out an experiment to identify genes (*cadD*) that confer resistance to cadmium antibiotics from bacterial plasmids. Subsequent investigations discovered these genes in five strains of MRSA isolates from Spain. In addition, the cadD gene for cadmium resistance was found in ST97, ST152, and ST8500, which together account for 44.74% of the isolates. While this study is among the few that has detected this gene in raw milk, another study conducted in the UK found it in animal feed at a detection rate of only 23% (Smith et al., 2005). Sequence Type 243 of the isolates of *S. aureus*, carried the gene *MurA*, which codes for resistance against fosfomycin. At different levels, fosfomycin resistance genes and mutations were found, and they were unmistakably linked to specific clonal complexes. According to Michalopoulos et al. (2011), the antibiotic fosfomycin targets the UDP-N acetylglucosamine enolpyruvyl transferase, which is involved in cell wall construction and encoded by the *murA* gene. Interestingly, the only strain with all the antibiotic resistance genes in this investigation was ST152, with the exception of the cadmium resistance genes. Quinolone, beta-lactam, ampicillin (*blaI, blaR1* and *blaZ*), and trimethoprim (*dfrG* and *dfrG 1*; *parE*) were also present in this ST152.

According to reports, beta-lactam antibiotics frequently cause staphylococci to exhibit resistance (Olsen et al., 2006; Zigo et al., 2022). The *blaZ* gene produces a penicillinase, also called a beta-lactamase, that imparts penicillin resistance by hydrolyzing the beta-lactam ring and rendering the drug inert (van den Borne et al., 2010; Zigo et al., 2022). Additionally, the results of this study support the conclusions that there is little to no *Staphylococcus* that is resistant to beta-lactam or penicillin (van den Borne et al., 2010). However, almost 45% of *S. aureus* isolates were found to be *blaZ* positive and phenotypically penicillin-resistant in a follow-up study conducted in New Zealand by Steele and McDougall (2014). This genotype/phenotype combination was associated with a very low cure following antibiotic treatment. The *mecA* and *mecC* genes were not present in any of the *S. aureus* isolates in the current investigation, which were all methicillin-sensitive *S. aureus* (MSSA). Furthermore, compared to a study by Haulisah et al. (2021) which revealed 80%–100% resistance to trimethropin, this study did not detect any of these genes from the isolates. Moreover, the production of toxins like hemolysins, leukotoxins, and enterotoxins as well as enzymes like serine proteases, cysteine proteases, and lipases that function as effectors during pathogenicity is what gives *S. aureus* its pathogenic potential (Sivakumar et al., 2023).

In addition to several enzyme-coding genes, the majority of the 38 *S. aureus* isolates included in this investigation carried multiple hemolysin genes. The genes encoding enterotoxins, enterotoxin-like proteins, and exfoliative toxins were found in just a small number of carefully selected genomes. Leukocidin D/E was found in all ST152 and ST342, while the Panton—Valentine leucocidin genes were only found in two sequence types (ST152 and ST243). Similar to this, all STs had higher frequencies of genes encoding adhesins and hemolysins, while isolates of *S. aureus* strains associated with bovine mastitis had lower frequencies of enterotoxins. Another crucial factor in determining virulence in staphylococci, particularly in *S. aureus*, is toxin synthesis. Inflammation and leukocyte cell death are promoted by these toxins, which include cytotoxins (hemolysins, leukotoxins, and leukocidins) and superantigens (enterotoxins, exfoliative toxins, and toxic shock syndrome toxins; TSST; Haag et al., 2019; Chen et al., 2022). In isolates from pus, skin infections, and abscesses, the *lukD* and *lukE* genes exhibited strong self-association. As their products are secreted prior to combining to create the PVL toxin, the genes *lukF-PV* and *lukS-PV* were associated in the current groups, which is consistent with the literature (Kaneko and Kamio, 2004; Rodrigues et al., 2022).

All *S. aureus* isolates in all clusters carried the cytotoxins *hlgA, hlgB*, and *hlgC*, which encode alpha, beta, and hemolysin, respectively. All *S. aureus* isolates used in the current investigation included leukocidin genes, such as ST97, ST352, *lukS-PV*, and *lukF-PV* (ST152 and ST243). This is consistent with research on bovine *S. aureus* isolates, which discovered leukocidin and leukotoxin genes in the majority of isolates (Wächter et al., 2021) from cows in India. This may explain similar observations from the current study because many of the *lukS-PV* and *lukF-PV* genes in our analysis shared the same percentage identity across different *S. aureus* isolates, suggesting that the gene sequences may be comparable to those of the genome even if they are absent. Many enterotoxin genes were also found in *S. aureus* isolates in this current study. The *aur* gene produces the protein aureolysin, which alters the adhesion factor *CflB* and triggers additional proteases to increase *S. aureus* pathogenicity (McAleese et al., 2001). The bi-component leukotoxins that are produced by the *hglA, hglB*, and *hglC* genes can create holes in cell membranes, which allows them to lyse cells (Staali and Colin, 2021). Since these gene products are linked to clinical mastitis, they could be valuable targets for the creation of vaccinations and therapeutic drugs (Ahmad-Mansour et al., 2021; Moawad et al., 2023).

The enterotoxins *sec, sei, sen, sem, seo*, and *seu* were also shown to be associated with similar frequencies in all analyzed groups; these findings have also been reported by other investigations (Indrawattana et al., 2013; Schwan, 2019; Ren et al., 2020). Additionally, prior research has demonstrated that *see* and *sec, sel*, are commonly found in MRSA strains (Hu et al., 2011); however, the current investigation did not find this association. It is crucial to prevent contamination of enterotoxin-producing *S. aureus* isolates throughout the food production chain since they can cause acute and severe food poisoning. Unpasteurized milk-based cheese and raw milk are well-known dietary sources of *S. aureus* food poisoning. It is believed that the chemotaxis inhibitory protein (*chp* product) and the *scn* gene product are highly specific for staphylococcal isolates of human origin (Pinchuk et al., 2010). The most typical cause of bovine mastitis is staphylococcal enterotoxin C. The primary enterotoxin gene of *S. aureus* isolated from cows with mastitis, according to other researchers, is called *sea*. Three enterotoxin-like genes have shown super-antigenic activity but no emetic qualities, two enterotoxin genes (*seg* and *sei*), and two enterotoxin genes make up the cluster (*selo, selm*, and *seln*). In the current investigation, 91.7% of the *S. aureus* isolates tested positive for the SEs coding genes classical expression (*sea, seb, sec*, or *sed*). The exfoliative toxins (*eta* and *etb*), toxic shock syndrome toxin-1 (tsst-1), staphylococcal enterotoxins (*sea, seb, sec, sed, saw, seg, seh, sei*, and *sej*), and *pvl* are all exoproteins that *S. aureus* is capable of producing (Ren et al., 2020). On the other hand, it is still unclear how staphylococcal enterotoxins affect mammary epithelial cells. Nineteen (19) serologically unique SEs have recently been found. Moreover, the genes most commonly found in *S. aureus* isolates from dairy cows with mastitis are *sec, sed, seg*, and *sei* and *sea* (Ren et al., 2020).

## 5 Conclusion

Understanding the epidemiology of *S. aureus* genotypes in dairy animals and herds may aid in developing treatment and control plans to stop the illness from spreading. Sequence Type 97, virulence genes like leucocidin, hemolysin, and aureolysin, and AMR genes like l*mrS*, *mepA*, and *tet (38)* were most frequently identified genes in this study. The capacity of *S. aureus* to colonize and penetrate the host may be influenced by the combination of these genes. Consequently, due to the global distribution of these genes, screening them in *S. aureus* isolates may be valuable for aiding in clinical outcome prediction and, specifically, for identifying hazardous strains. The current study also showed that all isolates had a nearly identical genotypic pattern and that certain isolates carried virulence factors such as *PVL-*encoding genes, suggesting that *S. aureus* isolates in animals should be closely monitored to prevent the spread of these genes. It is noteworthy that all isolates tested negative for *mecA* and *mecC*, Furthermore, the current study has also showed or revealed an association between ST97, *spa type* t2883 and t416 and the plasmid *Rep_N*. This study has shown that there is a wide range of *S. aureus* genotypes occurring in dairy cattle in the Free State province and that genetic variations are related to geographic origin of the isolates. This suggests that taking the region of interest and the strain virulence into consideration may help to formulate strategies directed to stop the spread of infection and to set up control measures in accordance with pathogen and host features. Hence, depending on the description of the circulating strain, the farmer would be able to choose whether to slaughter the sick animals or isolate positive cows using sanitary milking practices and an appropriate milking schedule.

## Data availability statement

The datasets presented in this study can be found in online repositories. The names of the repository/repositories and accession number(s) can be found at: https://www.ncbi.nlm.nih.gov/, PRJNA981445 and https://www.ncbi.nlm.nih.gov/, PRJNA1006054.

## Ethics statement

The animal studies were approved by Animal Research Ethics Committee of the University of Free State (UFS-AED2020/0060/21). The studies were conducted in accordance with the local legislation and institutional requirements. Written informed consent was obtained from the owners for the participation of their animals in this study.

## Author contributions

NK: Conceptualization, Data curation, Formal analysis, Funding acquisition, Investigation, Methodology, Resources, Writing – original draft. JN: Supervision, Writing – review & editing. ZM: Supervision, Writing – review & editing. SK: Formal analysis, Methodology, Software, Visualization, Writing – review & editing. OT: Supervision, Writing – review & editing.

## Footnotes

^1^ https://github.com/stanikae/jekesa

^2^ https://pubmlst.org/

^3^ https://github.com/tseemann/abricate

^4^ https://github.com/phac-nml/staramr

^5^ http://www.genomicepidemiology.org/services/

^6^ https://github.com/pha4ge/hAMRonization

^7^ https://github.com/tseemann/scapper

^8^ https://github.com/tseemann/snp-dists

^9^ https://microreact.org/

^10^ http://spaserver.ridom.de/
